# Research progress on quality assurance of genuine Chinese medicinal in Sichuan

**DOI:** 10.1186/s13020-021-00428-z

**Published:** 2021-02-08

**Authors:** Hua Luo, Yi Zhao, Hua Hua, Yan Zhang, Xiaobo Zhang, Qingmao Fang, Qingmiao Li, Yiguan Zhang, Peng Tan, Andong Yang, Shunyuan Jiang, Lanping Guo, Cheng Peng, Yitao Wang, Luqi Huang, Junning Zhao

**Affiliations:** 1 Institute of Chinese Medical Sciences, State Key Laboratory of Quality Research in Chinese Medicine, University of Macau, Macao, China; 2 Sichuan Institute for Translational Chinese Medicine, Sichuan Academy of Chinese Medical Sciences, Chengdu, China; 3 State Key Laboratory and Breeding Base of Dao-Di Herbs, Institute of Chinese Material Medica, China Academy of Chinese Material Science, Beijing, China; 4 College of Pharmacy, Chengdu University of Traditional Chinese Medicine, Chengdu, China

**Keywords:** Sichuan genuine Chinese medicinal, Quality assurance, Regionalization, Germplasm resources

## Abstract

The genuine Chinese medicinal (GCM), also known as Dao-di Herbs, is a synonym for high quality Chinese medicinal materials, which has been established in thousands of years of clinical practice and is a comprehensive standard for evaluating the quality of Chinese medicinal materials. The newest data from the Fourth National Survey of Chinese Medicinal Resources showed that Sichuan Province has 7290 types of Chinese medicine and 86 GCM, both ranking highly in China. The characteristics like diverse species, wide distribution, higher yield, and good quality are considered as advantages of geo-herbals grown in Sichuan. Resources guarantee and high-quality development of those medicine materials make a difference in local Chinese medicine quality promotion and Chinese medicine industry and technology development to serve the public's needs, assist targeted poverty alleviation, and strengthen ecological protection. This review aims to outline significant progress in the recent ten years regarding regionalization, germplasm resources, and quality evaluation around the quality assurance of GCM in Sichuan, China.

## Introduction

Genuine Chinese medicinal (GCM), referring to high-quality Chinese medicinal materials established by usage in clinical practice over thousands of years, functions as a comprehensive standard for Chinese medicine quality evaluation with historical and cultural attributes, and involves many influencing factors like genetics, environment, and production practice. From Han Dynasty to Ming-Qing Times, Sichuan Province, referring to related recording documents, was widely known as “the center of Chinese medicine and the warehouse of Chinese herbs,” imposing a great impact on traditional Chinese medicine for at least 2000 years. Among the 88 types of Chinese medicine materials recorded in the Zhou Dynasty’s earliest collection, “The Book of Songs,” 28 materials were native to Sichuan. By reading the “Laoguanshan medical book” (老官山医简), written in Chengdu’s Xi-Han dynasty, it was found that some medicine had been recorded, such as *Zanthoxylum bungeanum Maxim.* (ShuJiao, 蜀椒) and *Zingiber officinale* (ginger). The newest data from the Fourth National Survey of Chinese Medicinal Resources (FNSCMR) showed that Sichuan Province has 7290 types of Chinese medicine and 86 GCM, ranking highly in China. In recent years, Sichuan genuine Chinese medicine has been marketed at home and abroad for the characteristics of diverse varieties, wide distribution, higher yield, and excellent quality. Historically, pharmacies with traditional cultural connotation typically took genuine Chinese medicine from Sichuan, Guangxi, Yunnan as well as Guizhou, as selling points, and there is a saying goes, “no prescription is available without Sichuan genuine Chinese medicine” [1–3]. In the past 10 years, based on the FNSCMR, substantial progress has been made in research on production regionalization, germplasm resources, and quality evaluation, focusing on the quality assurance of Sichuan GCM. The relevant information is summarized as follows.

## Research on regionalization of Sichuan GCM

Resources of Chinese medicinal (CM) serve as a crucial material basis for the development of CM industry and traditional Chinese medicine (TCM), which also play a key role in strategic resources in China, supporting the inheritance and development of TCM culture. China has experienced national surveys of CM resources three times: the first one from 1960 to 1962 focused on commonly used CM; the second one was a national mass campaign of Chinese herbal medicine, investigating and collecting data on Chinese herbal medicine from 1969 to 1973 all over the country; from 1983 to 1987, the third survey of CM’s resources was completed by China National Medicinal Materials Corporation. Starting from 2011, the FNSCMR was organized by Luqi Huang, with the support of “3S” technology, including remote sensing (RS), geography information systems (GIS), and global positioning systems (GPS), as well as the computer network technology method, and digital photo technology, to more effectively collect location information of CM resources. It has provided data support for the future study on the differences and similarities of resources endowments, biological characteristics and spatial distribution patterns in regions [4, 5].

According to the latest data from the FNSCMR, Sichuan Province has 7290 kinds of CM resources and 86 kinds of GCM grown in Sichuan, making it one of the most important production areas of Chinese medicine materials. In 2017, the province artificially planted Chinese medicinal materials area about 4246.67 km^2^, of which 2206.67 km^2^ of *Eucommia ulmoides* Oliv., *Magnolia officinalis* Rehd. et Wils., and *Phellodendron chinense* Schneid. and medicinal materials were planted under forests. Among them, 31 materials are conserved by National Geographical Indications, and 16 varieties and 24 medicinal materials bases have been approved by Good Agricultural Practice for Chinese Crude Drugs (GAP). The approved 45 new varieties of Chinese medicinal materials including *Curcuma longa* L., *Ganoderma lucidum* (Leyss. ex Fr.) Karstand, *Aconitum carmichaelii* Debx., *Gastrodia elata* Bl., *Ligusticum chuanxiong* Hort., and *Carthamus tinctorius* L., etc., which number among the top in China. Based on these foundations, the seed-seedling bases and the germplasm resource base of Sichuan genuine Chinse medicine were built, and a dynamic monitoring system was established, while the observation points of CM were set up in key areas to dynamically monitor common and scarce species, and to realize the statistics aggregation and sharing of the census data from multiple sources.

Based on the latest survey data from CM resources (2011–2018), the long-term study of materia medica (from Han Dynasty to Qing Dynasty), and the integration innovation of "3S" information technology, a large-scale digital system research was conducted. With complete coverage, this research outcome determined the optimum distribution region, map, and area of Sichuan GCM, scientifically formulated the production area of 86 Sichuan GCM and achieved the regionalization of some cross-regional medicinal materials taking ecological and geographical environments into account. It provides new technical support for the high-quality GCM manufacture in Sichuan [6, 7]. From information of Table 1, following the principle of habitat adaptability, combined with factors such as landform, climate, and hydrology, the production area in Sichuan is divided into four regions, as shown in Table 1.

**Table 1:** The main ecological characteristics and the distribution of the four production areas

Project	Basin	Mountain area at the edge of basin	Chuan-Xi plateau and Chuan-Xi alpine gorge	Panxi district
Main Areas	Cities: Chengdu, Deyang, Mianyang, Ziyang, Meishan, Zigong, Neijiang, Suining, Nanchong, Guang'an	Cities: Yibin, Luzhou, Leshan,, Dazhou, Bazhong, Guangyuan,Yaan (Hanyuan county, Shimian county excepted)	Garze prefecture, Aba prefecture, Liangshan prefecture (Muli county excepted)	Liangshan prefecture (Muli county excepted), Panzhihua city, Ya'an city (Hanyuan county,Shimian county excepted)
Landform	Plain,hilly, mountain area	Hilly, mountain area	Alpine gorge and plateau	Mountain are, dry-hot valley, and hilly
Climatic zone	Subtropical humid climate	Subtropical humid vertical climate	Northern subtropical zone, temperate zone, frigid vertical climate	Southern subtropical climate, central subtropical climate
Annual average temperature(°C)	15–18	13–18	0–12	16–21
Annual accumulated temperature(°C)	4700–5200	4500–5000	600–4500	5600–7000
Annual accumulative irradiation time(hours)	890–1370	800–1400	2200–3000	3200–3300
Annual average precipitation(mm)	1000–1600	800–1800	520–890	700–1000
Main suitable varieties	*Ligusticum chuanxiong* Hort., Aconitum carmichaelii Debx., Ophiopogon japonicus (L. f), Angelica dahurica (Fisch. ex Hoffm.) Benth. et Hook. f, Pinellia ternata (Thunb.) Breit, Salvia miltiorrhiza Bge, Curcuma xvenyujin Y. H. Chen et C. Ling, Curcuma longa L., Alisma orientate (Sam.) Juzep., Paeonia lactiflora Pall., Carthamus tinctorius L., Chuanminshen violaceum Sheh et Shan, Houttuynia cordata Thunb., Psoralea corylifolia L., Citrus medica L. var. sarco dactylis Swingle, Gardenia jasminoides Ellis, Asparagus cochinchinensis (Lour.) Merr., Dendrobium nobile Lindl., Prunus mume (Sieb.) Sieb. et Zucc., Ginkgo biloba L. etc	Eucommia ulmoides Oliv., Magnolia officinalis Rehd. et Wils., Phellodendron chinense Schneid., Coptis chinensis Franch, Lonicera japonica Thunb., Gastrodia elata Bl., Cyathula officinalis Kuan, Platycodon grandiflorum (Jacq.) A. DC., Rheum palmatum L., Curculigo orchioides Gaertn., Aconitum carmichaelii Debx., Asparagus cochinchinensis (Lour.) Merr., Paris polyphylla Smith var. yunnanensis (Franch.) Hand. -Mazz, Bletilla striata (Thunb.) Reichb. f, Euodia rutaecarpa (Juss.) Benth, Fraxinus rhynchophylla Hance, Tremella fuciformis, Dipsacus asper Wall, ex Henry, Quisqualis indica L., Prunus mume (Sieb.) Sieb. et Zucc., Ginkgo biloba L., Stemona sessilifolia (Miq.) etc	Gastrodia elata Bl.、Rheum palmatum L., Fritillaria cirrhosa D.Don, Gentiana macrophylla Pall., Nardostachys jatamansi DC., Herpetospermum pedunculosum(Ser.)C.B.Clarke[Bryonia pedunculosa Ser, Gymnadenia conopsea(L.) R. Br., Notopterygium incisum Ting ex H. T. Chang, Angelica pubescens Maxim, f. biserrata Shan et Yuan, Eleutherococcus giraldii (Harms) Nakai, Rhodiola crenulata (Hook. f. et Thoms.) H. Ohb, Gentiana scabra Bunge, Moschus berezovskii Flerov etc	Paris polyphylla Smith var. yunnanensis (Franch.) Hand. -Mazz, Gastrodia elata Bl., Psoralea corylifolia L., Rheum palmatum L., Phellodendron chinense Schneid., Eucommia ulmoides Oliv., Dipsacus asper Wall, ex Henry, Panax notoginseng (Burk.) F. H. Chen, Aconitum carmichaelii Debx etc

It can be seen in Table 2 that on the basis of systematical summary combined with historical study and present regionalization situation of Sichuan GCM. Tons of information about Sichuan Province were collected and sorted, like the environmental factors: altitude, temperature, precipitation, and soil quality, the data about the recent remote sensing images from Digital Elevation Model and Enhanced Thematic Mapper, the information of land use and administrative district vector boundary, as well as the GIS environmental factors used to support overlay analysis of altitude, temperature, precipitation to get the optimum distribution district, map, and area of 86 GCM. At the same time, validation and correction were carried out according to the actual distribution of medicinal materials and was of benefit to the publication of "*Regional Plan for the Production of Genuine Chinese Medicines in Sichuan*" [8]. It is crucial for significantly strengthening resource protection and manufacturing management, effectively guiding the construction of production bases, rapidly promoting normalization and standardization, and steadily improving Chinese medicinal materials' quality.

**Table 2:** List of Genuine Chinese medicines in Sichuan (86 species)

No	Name							
1–8	*Croton tiglium* L	*Bletilla striata* (Thunb.) Reichb. f	*Paeonia lactiflora* Pall	*Angelica dahurica* (Fisch. ex Hoffm.) Benth. et Hook. f	*Paris polyphylla* Smith var. *yunnanensis* (Franch.) Hand. -Mazz	*Rheum palmatum* L	*Salvia miltiorrhiza* Bge	*Codonopsis pilosula* (Franch.) Nannf
9–16	*Pinellia ternata* (Thunb.) Breit	*Psoralea corylifolia L*	*Bupleurum chinense* DC	*Bufo bufo gargarizans* Cantor	*Cordyceps sinensis (*BerK.) Sacc	*Angelica pubescens* Maxim, f. biserrata Shan et Yuan	*Eucommia ulmoides* Oliv	*Citrus medica* L. var. *sarco dactylis* Swingle
17–24	*Citrus reticulata* Blanco	*Fritillaria cirrhosa* D.Don	*Paeonia veitchii* Lynch	*Melia toosendan* Sieb. et Zucc	*Aconitum carmichaelii* Debx	*Nardostachys jatamansi* DC	*Penthorum chinense* Pursh	*Zingiber officinale* Rose
25–32	*Chuanminshen violaceum* Sheh et Shan	*Clematis armandii Franch*	*Vladimiria souliei (*Franch.) Ling	*Cyathula officinalis* Kuan	*Ligusticum sinense* Oliv	*Pueraria lobata (*Willd.) Ohwi	*Uncaria rhynchophylla (*Miq.) Miq. ex Havil	*Cibotium barometz (*L.) J.Sm
33–40	*Iris tectorum* Maxim	*Aconitum carmichaelii* Debx	*Ligusticum chuanxiong* Hort	*Dipsacus asperoides* C. Y. Cheng et T. M. Ai	*Drynaria fortunei (*Kunze) J.Sm	*Lygodium japonicum* (Thunb.) Sw	*Polygonum multiflorum* Thunb	*Carthamus tinctorius* L
41–48	*Drynaria fortunei (*Kunze) J.Sm	*Lygodium japonicum* (Thunb) Sw	*Polygonum multiflorum* Thunb	*Carthamus tinctorius* L	*Acorus tatarinoxjuii* Schott	*Dendrobium nobile* Lindl	*Quisqualis indica* L	*Asparagus cochinchinensis* (Lour.) Merr
49–56	*Magnolia officinalis* Rehd. et Wils	*Polygonum cuspidatum* Sieb. et Zucc	*Zanthoxylum bungeanum* Maxim	*Phellodendron chinense* Schneid	*Trichosanthes kirilowii* Maxim	*Gastrodia elata* Bl	*Arisaema erubescens* (Wall.) Schott	*Tetrapanax papyrifer* (Hook.) K. Koch
57–64	*Polygonatum sibiricum* Red	*Coptis chinensis* Franch	*Astragalus membranaceus (*Fisch.) Bge. var. *mongholicus (*Bge.) Hsiao	*Curcuma longa* L	*Smilax glabra* Roxb	*Prunus mume* (Sieb.) Sieb. et Zucc	*Euodia rutaecarpa* (Juss.)Benth	*Rhus chinensis* Mill
65–72	*Tinospora capillipes* Gagnep	*Lysimachia christinae* Hance	*Lonicera japonica* Thunb	*Platycodon grandiflorum (Jacq.) A. DC*	*Amorphophallus rivieri* Durieu	*Paeonia suffruticosa* Andr	*Notopterygium incisum* Ting ex H. T. Chang	*Gentiana macrophylla* Pall
73–80	*Chrysanthemum morifolium* Ramat	*Ganoderma lucidum (*Leyss. ex Fr.) Karst	*Ophiopogon japonicus* (L. f)	*Buddleja officinalis* Maxim	*Fraxinus rhynchophylla* Hance	*Cornus officinalis* Sieb. et Zucc	*Moschus berezovskii* Flerov	*Cimicifuga heracleifolia* Kom

## Research on the Germplasm Resources’ Protection and Genetic Information of GCM Produced in Sichuan

Sichuan Province organized the high-quality germplasm resources of Sichuan GCM during the “Twelfth Five-Year Plan” period and collected more than 800 germplasm resources: 163 of unique germplasm resources such as *Lonicera japonica* Thunb., *Bupleurum chinense* DC., *Herpetospermum pedunculosum*, *Rheum palmatum* L., *Lamiophlomis rotata* (Benth.)Kudo, and *Trichosanthes kirilowii* Maxim.;186 of endangered germplasm resources such as *Gentiana macrophylla* Pall., *Rhodiola crenulat* (Hook.f.et Thoms.) H.Ohba, and *Paris polyphylla* Smith var. yunnanensis (Franch.) Hand. -Mazz; 249 of Sichuan germplasm resources, including, *Pinellia ternata* (Thunb.) Breit, *Aconitum carmichaelii* Debx., *Angelica dahurica* (Fisch. ex Hoffm.) Benth. et Hook. F, *Ligusticum chuanxiong* Hort., *Ophiopogon japonicus* (L. f). It collected and introduced 215 materials in 37 genera including *Ganoderma lucidum* (Leyss. ex Fr.) Karst, *Coriolus versicolor* (L. ex Fr.) Quel, *Morchella esculenta*, *Hericium erinaceus* (Rull ex F.) Pers., *Cordyceps militaris* and other fungus medicinal materials. Aiming at 24 genuine and special (fungus) medicinal materials produced in Sichuan, 35 new varieties have been selected and bred (Table 3). While selecting new varieties, each breeding unit has performed research on the growth and development characteristics, breeding methods, water and fertilizer management, field management, pest and disease resistance, and other cultivation techniques, and established relevant cultivation techniques to lay a foundation for new varieties breeding, demonstration, and extension, as well as industry development [9].

**Table 3:** Breeding of new varieties of traditional Chinese medicine in Sichuan

No	Medicine	Origin	Variety name
1	*Gastrodia elata* Bl	*Gastrodia elata* Bl. f. glauca S. Chow	Chuantianma Jinwu 1
2	*Salvia miltiorrhiza* Bge	*Salvia miltiorrhiza* Bunge	Chuandanshen 1
3	*Ganoderma lucidum* (Leyss. ex Fr.) Karst	*Ganoderma lucidum* (Leyss. ex Fr.) Karst	Yaolingzhi 2
4	*Perilla frutescens* (L.) Britt	*Perilla frutescens* (L.) Britt	Chuanzi 1
5	*Ligusticum chuanxiong* Hort	*Ligustricum chuanxiong* Hort	Lyuxiong 1
6	*Angelica dahurica* (Fisch. ex Hoffm.) Benth. et Hook. f	*Angelica dahurica* (Fisch. ex Hoffm) Benth. et Hook. f. var. formosana (Boiss) Shan et Yuan	Chuanzhi 2
7	*Cyathula officinalis* Kuan	*Cyathula officinalis* Kuan	Baoxi 1
8	*Salvia miltiorrhiza* Bge	*Salvia miltiorrhiza* Bunge	Zhongdan 1
9	*Gastrodia elata* Bl	*Gastrodia elata* Bl	Chuantianma Jinhong 1
10	*Ophiopogon japonicus* (L. f)	*Ophiopogon japonicas* (L. f) Ker-Gawl	Chuanmaidong 2
11	*Ligusticum sinense* Oliv	*Ligusticum sinense*. Oliv	Chenglong 1
12	*Curcuma phaeocaulis* Vai	*Curcuma phaeocaulis* Val	Chuanpeng 1
13	*Carthamus tinctorius* L	*Carthamus tinctorius* L	Chuanhonghua 3
14	*Ligusticum chuanxiong* Hort	*Ligusticum chuanxiong* Hort	Xinlyuxiong 1
15	*Iris tectorum* Maxim	*Iris tectorum* Maxim	Chuanshengan 1
16	*Aconitum carmichaelii* Debx	*Aconitum carmichaeli* Debx	Zhongfu 3
17	*Pinellia ternata* (Thunb.) Breit	*Pinellia ternate* (Thunb.) Breit	Chuanbanxia 1
18	*Penthorum chinense* Pursh	*Penthprum chinense* Pursh	Ganhuangcao 2
19	*Bupleurum chinense* DC	*Bupleurum scorzonerifolium* Willd	Chuanhongchai 1
20	*Bupleurum chinense* DC	*Bupleurum chinense* DC	Chuanbeichai 1
21	*Fritillaria cirrhosa* D.Don	*Fritillaria cirrhosa* D. Don	Chuanbei 1
22	*Trichosanthes kirilowii* Maxim	*Trichosanthes kirilowii* Maxim	Chuangualou 1
23	*Dendrobium nobile* Lindl	*Dendrobium denneanum* Kerr	Chuankehu 2
24	*Ganoderma lucidum (*Leyss. ex Fr.) Karst	*Ganoderma lucidum* (Leyss. ex Fr.) Karst	Yuze Lingzhi
25	*Ganoderma lucidum* (Leyss. ex Fr.) Karst	*Ganoderma lucidum* (Leyss. ex Fr.) Karst	Sanxiang Lingzhi
26	*Coriolus versicolor (*L. ex Fr.) Quel	*Coriolus versicolor* (L. ex Fr.) Quel	Yunzhi 1
27	*Curcuma longa* L	*Curcuma longa* L	Chuanjianghuang 1
28	*Curcuma phaeocaulis* Vai	*Curcuma phaeocaulis* Val	Chuanpeng 2
29	*Leonurus japonicus* Houtt	*Leonurus japonicas* Houtt	Chuanyi 1
30	*Gastrodia elata* Bl	*Gastrodia elata* Bl	Chuatianma Quanlyu 1
31	*Aconitum carmichaelii* Debx	*Aconitum carmichaeli* Debx	Zhongfu 4
32	*Coriolus versicolor (*L. ex Fr.) Quel	*Coriolus versicolor* (L. ex Fr.) Quel	Xianshan Yunzhi
33	*Polygonum multiflorum* Thunb	*Polygonum multiflora* Thunb	Panshouwu 1
34	*Dendrobium nobile* Lindl	*Dendrobium aurantiacum* Rehb. f. var. denneanum (Kerr) Z. H. Tsi	Lehu 1
35	*Lonicera japonica* Thunb	*Lonicera similis* Hemsl	Nanyin 1

Sichuan Academy of Chinese Medical Sciences took the lead in undertaking the projects about base construction of seed and seeding required for national essential drugs in 2012 and 2013 respectively, issued by the department of national traditional Chinese medicine, conducted the research to introduce conservation technology of medicinal plant resources, and collected 688 samples of 50 types of germplasm resources of Sichuan GCM. It is a nursery with the largest variety of GCM in China. Simultaneously, four regional seed and seedling bases (bases for storing seeds and seedlings of precious, special, genuine, endangered, bulk CM collected from the fourth resource census, studying the selection and breeding techniques of seeds and seedlings, and developing production technology standards, technology protocols, and setting quality standards) of Sichuan GCM were established, including Ya'an main base, Guang' an base, Emei Qiliping seedling base, and breeding bases for *Fritillaria cirrhosa* D.Don, *Aconitum carmichaelii* Debx., *Ophiopogon japonicus* (L.f), and *Ligusticum chuanxiong* Hort., which covered an area of more than 3.33 km^2^. Besides, systematic researches on breeding technology for *Fritillaria cirrhosa* D.Don, *Paris polyphylla* Smith var. yunnanensis (Franch.) Hand.-Mazz, *Notopterygium incisum* Ting ex H.T.Chang, *Ligusticum chuanxiong* Hort., *Curcuma longa* L., *Aconitum carmichaelii* Debx.and quality evaluation were carried out to form inspection test rules for *Notopterygium incisum* Ting ex H.T.Chang, *Ophiopogon japonicus* (L.f), and *Fritillaria cirrhosa* D.Don, set about 30 quality standards for seed and seeding, and formulate more than 30 rules of breeding and production technology, which filled the gaps in the seed and seedling standards of various Chinese medicinal materials in the southwestern region [3, 10]. In 2012, the state launched the National CM Germplasm Bank in Sichuan and Hainan. Chengdu University of Traditional Chinese Medicine constructed the Sichuan Bank. It passed construction acceptance in December 2017 and developed a preservation system with the long-term bank, medium-term bank, short-term bank, varieties nursery, separation bank, and DNA bank. It is planned to keep all of the germplasm resources collected in the FNSCMR, approximately 50,000 samples with 200,000 copies' storage capacity [11].

In recent years, with the advance of high-throughput sequencing technology, the research on medicinal plants' functional genomics has been greatly progressed. Some countries like Japan, the US and Germany have contributed their share in this field, studying on *Artemisia annua* L., *Glycyrrhiza uralensis* Fisch., *Catharanthus roseus* (L.) G. Don, *Taxus wallichiana* var. chinensis (Pilg.) Florin, and *Ginkgo biloba* L. In China, transcript sequencing and analysis of a batch of vital medicinal plants such as *Panax ginseng* C. A. Mey, *Panax quinquefolium* L., *Panax notoginseng* (Burk.), *Glycyrrhiza uralensis* Fisch., *Polygonum cuspidatum* Sieb. et Zucc., *Camptotheca acuminata* Decne., *Ginkgo biloba* L., and *Salvia miltiorrhiza* Bge have been completed. Nevertheless, in general, due to the late start of identifying functional genomics of medicinal plants, some rare medicinal plants' genetic background is not clear, the genetic information is inadequate, and the essential data is less. Information in Table 4 demonstrates that the transcript studies of Chinese medicinal materials and natural medicines have been completed mainly in China, India, Japan, South Korea, and Canada, with single-variety small sample sizes.

**Table 4:** Name of transcript sample of medicinal plant and related research groups

Medicinal plants’ name	Latin names	Research group	Time
*Cannabis sativa* L	*Cannabis sativa*	Timothy R Hughes, Jonathan E Page	2011
*Astragalus propinquus Schischkin*	*Astragalus membranaceus* Bge. var. *mongolicus* (Bge.) Hsiao	Xuan Li, Peng Nan	2015
*Gnetum parvifolium*	*Gnetum*	Zeping Jiang, Shengqing Shi	2016
*Cistus ladanifer* L	*Cistus creticus subsp. creticus*	Angelos K. Kanellis	2008
*Hippophae rhamnoides Linn*	*Hippophae rhamnoides* L	Priti Krishna; Prakash Chand Sharma; Prakash Chand Sharma; Prakash Chand Sharma	2012; 2012; 2013; 2014
*Picrorhiza scrophulariiflora*	*Picrorhiza kurrooa* Royleex Benth	Ravi Shankar, Sanjay Kumar	2012
*Salvia miltiorrhiza Bunge*	*Salvia miltiorrhiza*	Shilin Chen; Wang Zhezhi; Changqing Yang; Xiu-Jie Wang, Reuben J Peters, Luqi Huang; Xingfeng Li	2010; 2011; 2013; 2014; 2017
*Physalis peruviana* L	*Physalis peruviana*	Leonardo Mariño-Ramírez	2012
*Dendrobium officinale Kimura et Migo*	*Dendrobium officinale* Kimuraet Migo (Orchidaceae)	Shilin Chen	2013
*Withania somnifera*	*Withania somnifera*	Parul Gupta	2015
*Sophora flavescens var. flavescens*	*Sophora flavescens*	Kazuki Saito	2015
*Fallopia multiflora*	*Polygonum cuspidatum*	HAO DaCheng, CHEN ShiLin	2015
*Hypericum perforatum*	*Hypericum perforatum*	Zhezhi Wang	2012
*Carthamus tinctorius*	*Carthamus tinctorius* L	Hu Shangqin	2012
*Panax quinquefolius*	*Panax quinquefolius* L	Shilin Chen; Dan Brown; Tae-Jin Yang	2010; 2013; 2014
*Benincasa pruriens*	*Benicasa hispida*	Dasen Xie	2013
*Lycium chinense*	*Lycium chinense*	Ying Wang	2015
*Plantago ovata* FORSK	*Plantago ovata*	Sanjana Kaul	2016
*Azadirachta indica*	*Azadirachta indica* A.Juss (neem)	Binay Panda	2012
*Calotropis gigantea* (L.) Dry.ex Ait.f	*Calotropis procera* R. Br	Pahn-Shick Chang	2015
*Taxus chinensis*	*Taxus chinensis*	Long-jiang Yu	2012
*Ganoderma lucidum*	*Ganderma lucidum*	An-Yuan Guo, Xingyao Xiong	2012
*Hypericum perforatum*	*Hypericum perforatum*	Zhezhi Wang	2012
*Panax quinquefolius*	*Panax quinquefolius* L	Shilin Chen; Dan Brown; Tae-Jin Yang	2010; 2013; 2014
*Benincasa pruriens*	*Benicasa hispida*	Dasen Xie	2013
*Lycium chinense*	*Lycium chinense*	Ying Wang	2015
*Plantago ovata* FORSK	*Plantago ovata*	Sanjana Kaul	2016
*Azadirachta indica*	*Azadirachta indica* A. Juss (neem)	Binay Panda	2012
*Calotropis gigantea (L.) Dry.ex Ait.f*	*Calotropis procera* R. Br	Pahn-Shick Chang	2015
*Taxus chinensis*	*Taxus chinensis*	Long-jiang Yu	2012
*Paris polyphylla*	*Paris polyphylla* Smith var. yunnanensis (Franch.) Hand. -Mazz	Shengchao Yang	2016
*Poria*	*Wolfiporia cocos*	Haiyang Xia, Mo Wang	2013
*Trillium govanianum*	*Trillium govanianum*	Ram Kumar Sharma	2017
*Bupleurum*	*Radix bupleuri*	Jianhe Wei, Shilin Chen	2014
*Rehmannia glutinosa*	*Rehmannia glutinosa*	Xianen Li; Fengqing Wang	2012; 2017
*Houttuynia cordata*	*Houttuynia cordata* Thunb	Xianjin Wu	2014
*Taxus*	*Taxus cuspidata*	Shilin Chen	2011
*Digitalis purpurea*	*Digitalis purpurea*	Shilin Chen, Shanfa Lu	2012
*Swertia mussotii*	*Swertia mussotii* Franch	Yue Liu, Yi Wang	2017
*Polygonum muricatum*	*Polygonum minus*	Hoe-Han Goh	2017
*Panax notoginseng*	*Panax notoginseng* (Burk) F.H. Chen	Shilin Chen	2011
*Andrographis paniculata (Burm. f.) Nees*	*Andrographis paniculata*	Dashavantha R.Vudem	2016
*Papaver somniferum* L	*Papaver somniferum*	Prabodh Kumar Trivedi	2013
*Mucuna pruriens*	*Mucuna pruriens* (L.) DC	N.Sathyanarayana, Ashley N. Egan	2017
*Ophiocordyceps sinensis*	*Ophiocordyceps sinensis*	Shilin Chen	2014
*Millettia speciosa* Champ	*Callerya speciosa* (Champ.) ScHot	Zhiying Li	2016
*Epimedium brevicornu Maxim*	*Epimedium sagittatum* (Sieb.EtZucc.) Maxim	Ying Wang	2010
*Macleaya cordata* (Willd.) R. Br	*Macleaya cordata and Macleayamicrocarpa*	Jianguo Zeng, An-Yuan Guo, Xingyao Xiong	2013
*Rosa banksiae* var. normalis	*Aquilaria sinensis* (Lour.) Gilg	Jianhe Wei	2012
*Raphanus raphanistrum subsp. sativus*	*Raphanus sativus*	Maoteng Li	2013
*Rhodiola algida*	*Rhodiola algida* L	Shilong Chen	2014
*Gentiana scabra*	*Gentiana rigescens*	Yuanzhong Wang	2015
*Amorphophallus konjac*	*Amorphophallus*	Ying Diao, Zhongli Hu	2013
*Angelicae sinensis radix*	*Angelica sinensis*	Lili Niu	2016
*Paeonia ostii T. Hong et J. X. Zhang*	*Paeonia suffruticosa cv.* FengDan	Luqi Huang	2017
Sichuan genuine Chinese medicines	100 types of medicinal plants in Sichuan	Junning Zhao	2020

Sichuan Academy of Chinese Medical Science and BGI (Beijing Genomics institution) college, cooperated closely to collect and select Sichuan GCM with high clinical value in different growth phases, medicinal parts, locations, and species, employing Illumina HiSeq2000 High-throughput sequencing technology. It is the first time to conduct large-scale transcript research (100 T-SGH, Transcript study of hundreds of Sichuan genuine herbs) at home and abroad on hundreds of Sichuan GCM and big brand CM (refers to CM with significant or exact clinical efficacy, meeting clinical needs, high technological content, and occupying a large market share), establish a unified experiment procedure: sample processing, RNA extraction, library construction, and sequencing, and explain the molecular mechanism of high-quality Sichuan GCM with a comparison of sequencing quantity, assembly length, assembly results, the ratio of annotation to KEGG, Species Tree and other parameters, which are all at a maximum level around the world [3, 12, 13].

## Quality assessment technology and quality assurance of Sichuan GCM

GCM pharmacology was first proposed to objectively describe the drug effects of GCM, scientifically explain the related mechanism, explore the standards and methods based on biological effects and clinical efficacy to offer scientific evidence for drug property theory, pharmacological mechanism, and clinical treatment. A function of the Microtox (micro-toxic) technology with independent intellectual property rights is to carry out pharmacological and bio-quality evaluation technology research on Sichuan GCM's extracts. The objective is to realize standardization, speedy, quantitative characterization of biological effect (toxic) value, toxic dose–effect curve, as well as biological fingerprints of CM, and set up a new bio-control model and assessment system closely related to clinical efficacy, revealing the complex properties like diverse components, various targets, multiple effects of CM, in an overall view. The system provides ample data and scientific support for CM's quality control and safety supervision [14–20]. Also, as a representative Sichuan genuine Chinese medicine, *Aconitum carmichaelii* Debx., it was characterized by diverse germplasm resources, complex chemical composition, multi-directional pharmacological effects and wide clinical applications. Hence, a method of "multi-dimensional evaluation and integrated analysis" was brought up with the guidance of "systemic CCM", to systematically study the "quality, properties, process, efficacy, and use" of *Aconite.* This method can also objectively describe the relationship between *Aconite's* toxicity and efficacy to interpret the complex association between drug and body, and provide a scientific basis for the quality evaluation and rational application of *Aconite* produced in Sichuan, China [21].

**Fig. 1:**
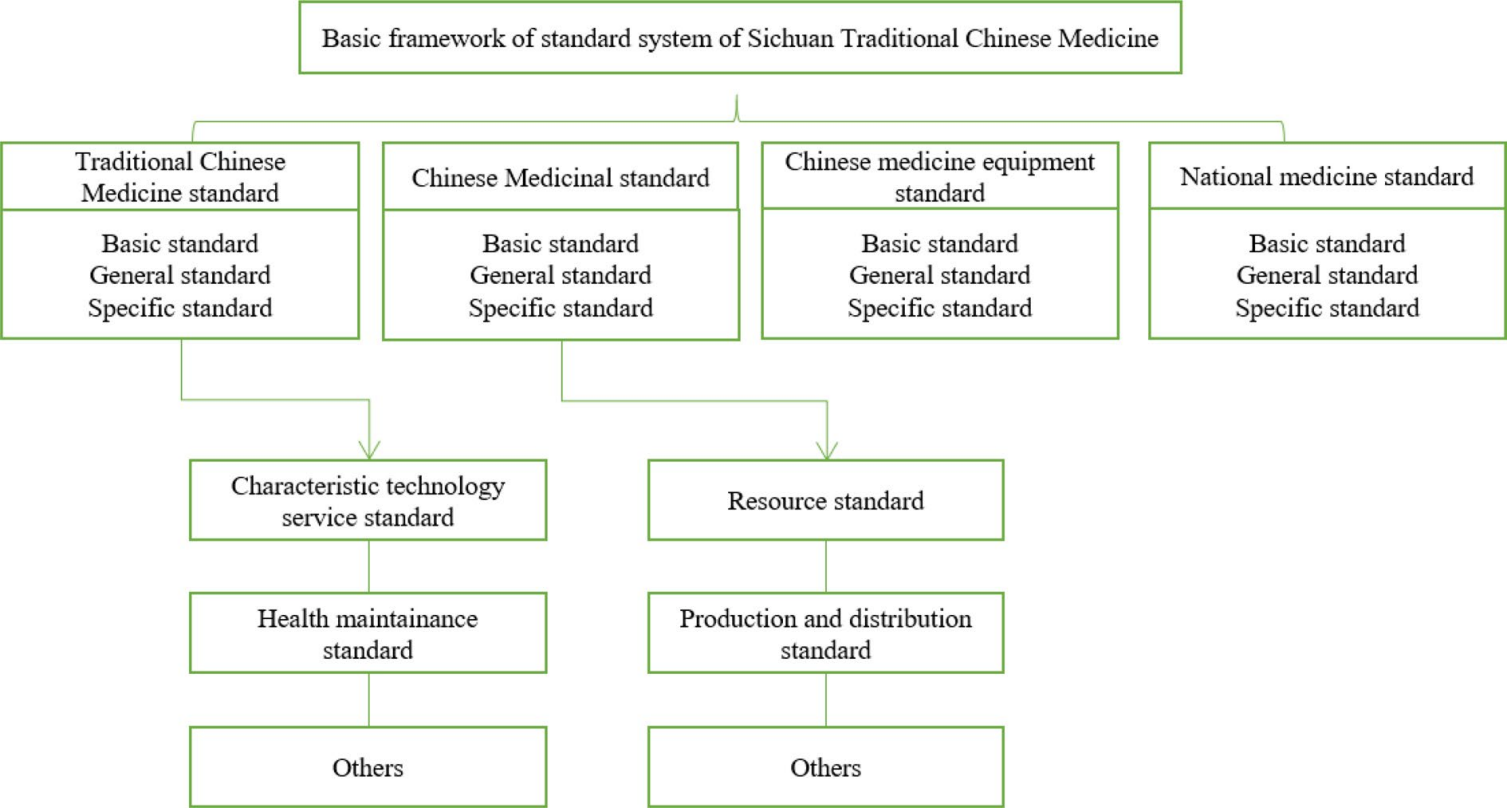
Basic construction framework of standard system of Sichuan genuine Chinese medicinal

**Fig. 2:**
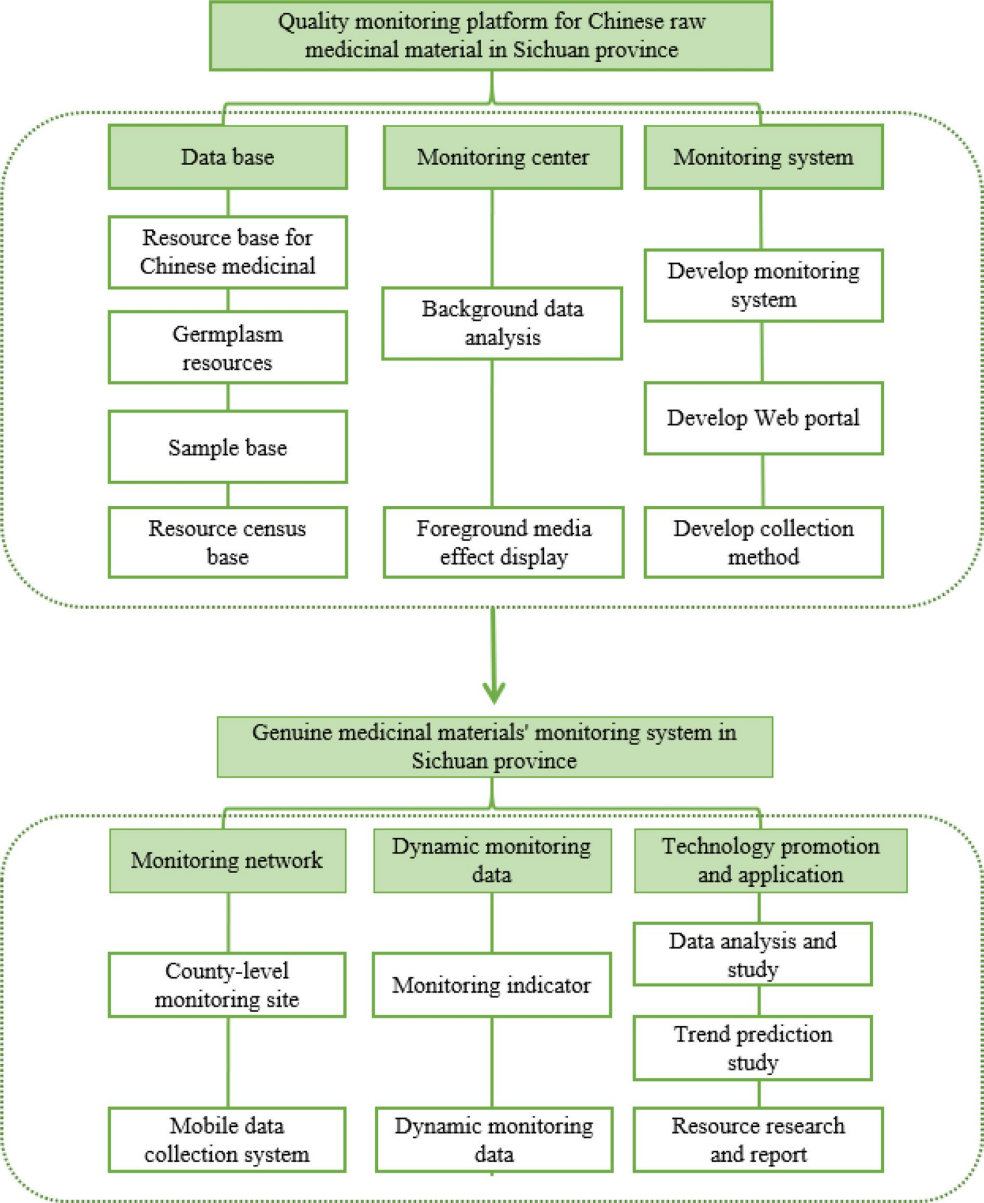
Dynamic monitoring system of Sichuan genuine Chinese medicinal

The framework in Fig. 1. shows that a standard system of Sichuan GCM is outlined and develops a series of medicinal material standards. Relatively, the local standards, and the group standards issued by the Chinese Association of Chinese Medicine concerning the "*Commodity Grades of Chinese Medicinal Materials*" and "*Technical Rules for Cultivation and Production areas of Genuine Chinese Medicinal Materials*" have been implemented, referring to Table 5 [22]. Subsequently, a dynamic monitoring system and a comprehensive information platform have been constructed, showed in Fig. 2, in which the study on the dynamic monitoring system is the core part, and the construction of an integrated information platform is the goal. Equally, this work involves the three-level combination of monitoring center, monitoring sites, and monitoring spots, combined with big data analysis and visualization technology.

**Table 5:** Local standards (DB) of Sichuan Province's first batch of genuine Chinese medicines in 2018

No	Name of local standard	Serial number of local standards
1	General principles for certification of Sichuan genuine Chinese medicines	DB51/T 2565–2018
2	Certification of Sichuan genuine Chinese medicines -soil quality control	DB51/T 2559–2018
3	Sichuan genuine Chinese medicines ' certification of Curcuma longa L	DB51/T 2561–2018
4	Sichuan genuine Chinese medicines ' certification of *Ligusticum chuanxiong* Hort	DB51/T 2562–2018
5	Sichuan genuine Chinese medicines ' certification of *Codonopsis pilosula* (Franch.) Nannf. (Jiuzhai)	DB51/T 2563–2018
6	Sichuan genuine Chinese medicines ' certification of Notopterygium incisum Ting ex H. T. Chang	DB51/T 2564–2018
7	Sichuan genuine Chinese medicines ' seeding assortment of Ophiopogon japonicus (L.f)	DB51/T 2557–2018
8	Sichuan genuine Chinese medicines' production technique rules for *Angelica dahurica* (Fisch. ex Hoffm.) Benth. et Hook. f	DB51/T 2558–2018
9	Sichuan Genuine Chinese medicines ' production technique rules for *Aconitum carmichaelii* Debx	DB51/T 2560–2018
10	Sichuan Genuine Chinese medicines ' production technique rules for *Salvia miltiorrhiza* Bge	DB51/T 2566–2018

The establishment of the resource network system and dynamic monitoring operation of CM resources can guide the cultivation of Chinese medicinal materials, ensure market supply, and improve the quality of medicinal materials [3, 10]. Study on the quality assurance and traceability system of Chinese medicinal materials based on the integration of the whole industrial chain standard (means completing the product design, storage and transportation, raw material procurement, order processing, wholesale operation, and terminal retail of the whole industry chain with higher efficiency, so as to take the initiative and lead in market adaptation and consumer interaction and achieve the purpose of efficient integration), with the 7S quality assurance and management system (including the identification of GCM, germplasm selection and standardized planting, fidelity standardized herb processing, fidelity standardized testing, fidelity standardized packaging, fidelity constant control storage, fidelity full traceability), established the data standards and relation data model for whole process quality control of Chinese medicinal materials. It also applied information technologies, including the Internet of Things, the Internet and blockchain to establish dynamic monitoring and control system, production management system, and whole process quality traceability system to ensure production management, data collection, storage and traceability of the whole process of Chinese medicinal materials more effectively. The system was recorded by the China Certification and Accreditation Administration of the P.R.C in 2018 and currently it has become a certification standard of quality management system for the CM industry [23].

## High-quality development countermeasures of Geo-authentic medicinal materials produced in Sichuan

Chinese medicinal materials are the basis for the inheritance and development of TCM and are strategic resources to improve the national economy and people's livelihood. The Sichuan region spans the Qinghai-Tibet plateau, the Hengduan mountains, the Yunnan-Guizhou plateau, the Qinba mountains, and the Sichuan basin. The terrain is high in the west and low in the east, and slopes from the northwest to the southeast, involving four types of landforms like plain, hill, mountain, and plateaus. Therefore, it is not only an important water conservation area in the Yellow River and the upstream of the Yangtze River and but also a treasure house of biodiversity in western China. As far as Sichuan GCM are concerned, there are some relevant issues: a. The production layout lacks scientific consideration, and the planting production is not standardized; b. The planting base infrastructure is backward, and the scientific and technological innovation lacks effective integration; c. The slow pace of integrating big brand CM with big industry has become a significant problem that impedes the conversion from resource advantages into market power and regional economic strength in a more efficient way. Thus, to better the high-quality development of Sichuan GCM, some breakthroughs in ideas and methods should be made as soon as possible.

First and foremost, it is necessary, based on the theory of "General CM," to integrate the relevant elements, like TCM theory, clinical treatment, comprehensive exploitation, industry advance, health services, resource preservation, ecological environment, and culture inheritance, to construct a technology platform for systematic research and development of Sichuan GCM (GCM-SRD platform), as well as around the three critical links of the production system, standard system, and traceability system. The aim is to equip central technical units with standardized production, chemical and quality study, drug efficacy and quality control, product development and pilot testing, and to provide systematic, complete, engineered solutions and open services for the cultivation of big brand of GCM and the boost of the health industry [24].

According to the suitable growth requirements of *Fritillaria unibracteata* Hsiao et K. C. Hsia, a Sichuan GCM, Qingmao Fang et al. obtained the data of land use status through remote sensing and GIS spatial analysis. In addition, combined with the quantitative and comprehensive analysis of the environmental indexes for the growth of *Fritillaria unibracteata* Hsiao et K. C. Hsia, the suitable distribution range was found. Moreover, the results demonstrated that Hongyuan, Songpan, Ruoergai, Jiuzhaigou, Maoxian, Heishui, Lixian, Pingwu, Maerkang and other areas in Sichuan province were the suitable distribution areas. Among them, 7 counties including Hongyuan, Songpan, Ruoergai, Maoxian, Heishui, Maerkang and Jiuzhaigou were the main distribution areas of *Fritillaria unibracteata* Hsiao et K. C. Hsia, accounting for 45.2% of the suitable areas. In addition, the result of the field investigation of the resources of *Fritillaria unibracteata* Hsiao et K. C. Hsia, was consistent with the study’s results [7]. A recent study of *Fritillaria cirrhosa* D. Don., using properties observation, thin layer chromatography (TCL), and content determination methods to conduct a detailed description, and found that there was a huge difference between cultivated and wild *Fritillaria cirrhosa* D. Don. in properties. In detail, the leaf of cultivated one was yellow, slightly rough, and shrivelled, while Qingbei (青贝) was bigger (up to 4 cm). Among the cultivated products in the market, those with the characteristics of Songbei (松贝) mainly came from the *F.unibracteata* Hsiao et K.C.Hsia, while those with the characteristics of Qingbei (青贝) mainly derived from the *Fritillaria cirrhosa* D.Don, *Fritillaria unibracteata* var. wabuensis and *F.taipaiensis* P.Y.Li. Besides, the TLC characteristics of *Fritillaria cirrhosa* D.Don were mainly related to plant origins, while the effective components had similar structures but different subtle structures. Additionally, the total alkaloid content between cultivated products with characteristics of Songbei and with characteristics of Qingbei were varied [25]. Another research systematically analyzed the differences between the cultivated materials of Fritillaria dulcinea and Fritillaria Warb to provide the basis for the accurate evaluation of the quality of them. Researchers systematically analyzed and compared the cultivated materials of 8 batches of *F.unibracteata* Hsiao et K.C.Hsia and 12 batches of *Fritillaria unibracteata* var. wabuensis in terms of the appearance, content and TCL characteristics. Finally, the results showed that the differences existed in appearance traits, composition content and TLC characteristics [26].

Another one is to build a genetic data platform for Sichuan GCM. In 2009, the Thousands of Plants Transcript Project (https: / /www. onekp. com/) jointly initiated by scientists from the United States, Canada, and China, plans to complete the transcript sequencing of 1,000 plants. This amount covered most of the plant families, by far the largest genetic resources program of plant. As of October 2018, the program has finished sequencing, archiving, and data analysis of more than 1,400 species. The research of GCM produced in Sichuan is a particular field requiring comprehensive analysis with data such as genetics, metabolism, and environmental elements. Such an extensive research will rely more on multidisciplinary experts' joint contributions and require different perspectives on the same data. Since 2017, Junning Zhao’s team of Sichuan Academy of Chinese Medicine Sciences have cooperated with BGI college to conduct large-scale transcript research on 300 samples of 100 varieties of Sichuan GCM, laying the foundation for the genetic data platform establishment and effectively supplementing the data of existing medicinal plants' genetic resources. In addition, only by comparing the genetic resource of medicinal plants with plants' data can new discoveries be made on a broader level of vision [12].

Furthermore, it is crucial to formulate scientific standards for Sichuan GCM. This measure needs to follow the characteristics of TCM and the natural growth law of medicinal materials, accelerate the construction of the standard system of Chinese medicinal materials in Sichuan, establish the production standards, product standards, processing standards, and storage standards more systemically, initiate and promote the certification of restorative materials, build the third-generation CM traceability system based on blockchain, Cloud and Big data, and the service platform management system for the quality traceability of CM industry to promote the formulation of domestic and international market pricing standards that reflect quality first and benefit priority orientation [14, 22].

Lastly, one solution is to break through the bottleneck of high-quality technology and perfect the relevant equipment of Sichuan GCM. If we expect to achieve this goal, it is urgent to speed up interacting GCM with advanced technology like 5G communication and blockchain. This method can not only raise the intellectual level of the system about production, quality, and traceability, but also strengthen the innovative technology in protecting germplasm resources, setting up seed and seedling bases, and planting ways, playing a leading role in technology and equipment upgrade [27].

On the basis of whole industrial chain standards, Deng Bin et al. integrated the technical platform of CM’s quality assurance and 5G communication technology traceability system, in which the 7 management requirements involving planting (breeding), harvesting, processing, packaging, testing, storage and quality traceability management were constructed, to form a new quality management system certification standard for GCM. This achieved a seamless connection between the origin of GCM such as *Ophiopogon japonicas* (L.f) Ker-Gawl., *Salvia miltiorrhiza* Bunge, *Fritillaria cirrhosa* D. Don., and *Curcuma longa* L., and the consumer market to implement the whole process of quality fidelity control from the source [23].

## Conclusion and perspectives

To sum up, this article focuses on explaining some critical technologies about the research and development of Sichuan GCM, such as genetic information and formation mechanism, quality biological evaluation, and cultivation of large varieties. Furthermore, based on the latest results of the FNSCMR in Sichuan Province, we systematically summarize the related research history, the current status of regionalization, the germplasm resources, the quality evaluation, and the quality assurance to greatly help researchers make a technical breakthrough in data resources analysis, high-quality technical equipment modification, new models establishment for high-value development of characteristic resources of genuine Chinese medicine, and to boost the inheritance and innovation of TCM.

## Data Availability

Not applicable.

## Abbreviations

CM: Chinese medicinal
FNSCMR: Fourth National Survey of Chinese Medicinal Resources
GCM: Genuine Chinese medicinal
GIS: Geographic Information System
GPS: Global Positioning System
KEGG: Kyoto Encyclopedia of Genes and Genomes
RS: Remote sensing
TCM: Traditional Chinese Medicine
T-SGH: Transcript study of hundreds of Sichuan genuine herbs
GCM-SRD: Genuine Chinese medicinal for systematic research and development
